# Evaluation and Analysis of Quantitative Architectural Space Index Based on Analytic Hierarchy Process

**DOI:** 10.1155/2022/4911589

**Published:** 2022-03-09

**Authors:** Congxiang Tian, Xiancheng Liu, Yong Yang, Guoqing Zhu

**Affiliations:** ^1^Yangtze University College of Arts and Sciences, Jingzhou 434100, China; ^2^School of Urban Construction, Yangtze University, Jingzhou 434100, China

## Abstract

With the continuous development of the social economy, the urban residential structure is also changing, and people have higher and higher requirements for the living environment. Moreover, the landscape construction of public spaces in cities is an important part of the city. It is easy to neglect the comprehensive consideration of historical development and regional culture in architectural projects. The overall lack of individuality in urban design, the lack of characteristics of adapting measures to local conditions, and the blind emphasis on architectural landscaping have led to a serious lack of regional cultural characteristics and spiritual culture in public places. Therefore, in terms of the problems of insufficient landscape construction quality and green concepts being insufficient in the construction environment of cities, an evaluation method system is established in the paper to further study the shortcomings of the scenic area architecture. What is more, relying on the personal experience of the users in the scenic spot and understanding the real needs of the public space landscape environment around the scenic spot, scientific methods and a complete system are applied to make an overall assessment of the public space in the built residential area. Besides, according to the data analysis of the simulation experiment, the advantages and disadvantages of the public space in the residential area are extracted. Combined with the current research status, the green concept design strategy of the public space landscape environment in the scenic spot is summarized. Lastly, according to the data analysis of the simulation experiment, the evaluation satisfaction of the activity atmosphere, plant configuration, and overall layout is improved by 4.2%, 3.7%, and 3.1% compared with other methods, which proves that this study has a more reasonable planning program to meet the various needs of public open space. The development of urban landscape design provides a valuable reference.

## 1. Introduction

The design and construction of public spaces in cities is an important issue that needs to be seriously addressed in future urban construction. After the rapid development of urban construction in the past, a series of urban problems have gradually become prominent, such as a lack of cultural atmosphere, unreasonable humanization of construction, and non-energy-saving and environmental protection [1]. Nowadays, the planning and construction of cities are in the process of changing from quantity to quality and improving the quality of the city has become the focus of the whole society. Urban public life is closely related to urban public space. Therefore, based on the landscape construction area, public space is not only a place for residents' daily life communication but also an important urban space for urban residents' activities [2]. It is of great significance to build a good urban atmosphere, which will enrich the urban culture and create a scenic environment with a green concept [3].

In 2009, David Fletcher expressed in his paper “Landscape Urbanism and the Los Angeles River” that in the process of urban construction. The understanding of ecology should not only consider the external conditions of the city but also analyze the relationship between the environmental atmosphere and the physical location of the city, which is guided by the concept of landscape urbanism and takes the Los Angeles River as its object [4]. A series of strategies such as traffic flow lines, water area restoration, and urban development in urban open space are explored. With the development of landscape urbanism, more and more views of landscape urbanism have been applied to large-scale urban waterfront renovation projects, and many positive feedbacks have been received. In the early stages, there were waterfront renovation projects in Toronto, Canada, water planning in Philadelphia, New York, and Fleiss Landfill Park in New York [5]. The design of Japan International Yokohama Pier and Elwood Beach is a successful classic case in recent years. For example, in the design of Elwood Beach, the designer connects different types of urban spaces through the arrangement of parallel landscape elements, which solves the problem of a lack of interaction between beaches and public spaces [6].

The research object of the paper is the public space landscape design of the scenic spot, namely the square, street, green space, and other different forms of activity space in the scenic spot, and related supporting facilities and cultural characteristics. Data indicators are mainly quantified in the overall structure, planning layout, openness of the scenic spot, and the usage of public space in the landscape environment. Meanwhile, through the postuse evaluation, the overall situation is comprehensively scored, and the existing problems and advantages are analyzed and summarized to put forward rectification suggestions, and finally explore the design strategy of the scenic public space landscape with certain regional adaptability.

The main study innovations in the paper are as follows:

1. Establishing an evaluation index of the architectural landscape to realize the objective evaluation of the architectural landscape by quantifying the index.
2. Using the analytic hierarchy process to sum up the architectural landscape index as the research scheme to ensure the evaluation quality.
3. Establishing the decentralization of landscape architecture quality evaluation elements to improve the fitness of evaluation.

## 2. Functional Layout of Building Indicators

According to the architectural layout, the spatial layout is formulated in combination with functional facilities.

(1) According to the different nature of space, the space is divided into three levels: public space, semipublic space, and private space. Secondly, in the semipublic space, through the natural division of roads, four groups of similar scale are formed in the residential area so that the people in the residential area can be reasonably diverted. The central green space of the group and the public facilities around the road interface of the group are dotted [7].
(2) At the level of group space, open residential areas form a number of courtyards through the enclosure of buildings. In courtyard space, as the space closest to the privacy of residents' lives, in principle, there is no intervention of public service functional buildings. The scale is strictly controlled. The space's character is restrained and independent, and the living environment needs of quiet homes are protected. The three levels of space are progressive in their planning, which not only ensures the openness and interoperability of residential space but also takes into account the integrity of space experience and the feeling of psychological safety of residential areas.
(3) Road traffic
  ① The roads in the residential area are vertical and horizontal in structure. The roads at the residential area level are the main channels for residents to travel.
  ② The district-level road connects the central green space of each group. Some sections are designed as tree-lined roads, on both sides of which public service facilities are arranged on the ground floors of residential buildings. In the planning, road traffic organization is incorporated into the environmental design of residential areas.
(4) Supporting facilities for shared public buildings
Because of the open planning, the supporting facilities of public buildings in residential areas are based on the principle of full sharing. The open commercial space of the residential area realizes the life transformation of the urban interface of the residential area. Residential commerce is divided into the following two levels:
① The commerce at the central level of the residential area has a relatively certain scale and is set up in a unified way with the planned Du District Center;
② Neighborhood level businesses in the residential area are located on the ground floor of the residential area along the pedestrian system of the residential area, with flexible distribution.

## 3. Quantitative Evaluation of the View of a Building Based on Green Concept Landscape

### 3.1. Selection of Evaluation Indicators

As an important content of the evaluation, the evaluation index should meet the needs of the residents to the greatest extent, which should also be reasonable and easy to operate. According to the practical research of domestic and foreign scholars, after soliciting opinions from internal and external sources, and discussing with the representatives of design, construction, property units, and owners, the indicators that affect the satisfaction of occupants are finally summarized [8].

Six first-level indicators are planning layout, road traffic, activity space, green space, supporting facilities, and humanistic spirit. In addition, 24 second-level indicators are refined according to the first-level indicators, as shown in Table 1.

### 3.2. Establish an Evaluation Index System

**Figure 1:**
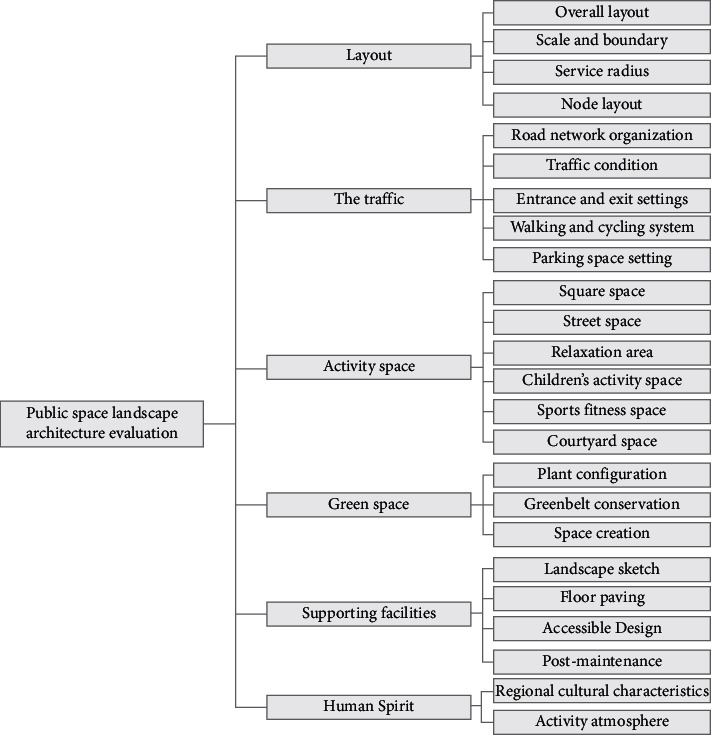
Evaluation factors of public space landscape architecture.

The following principles should be followed when constructing the evaluation index system:

1. In general, the evaluation factors are divided into three levels, namely the target level, the criterion level, and the plan level.
2. Factor concepts at the same level should not interfere with each other and try to avoid similar factors.
3. The number of evaluation factors under the same level branch is preferably no more than 9 so that the quality of the sample can be ensured.
4. The number of factors at the last level does not exceed 25 at most.
5. The selected evaluation index factors are added according to the target level, criterion level, and program level to establish the evaluation index system [7].
6. Target level: public space landscape evaluation.
7. Criterion level: It is composed of six first-level evaluation index factors: planning layout, road traffic, activity space, green space, supporting facilities, and humanistic spirit.
8. Scheme level: the first category is planning layout, which is composed of four secondary evaluation indicators: overall layout, scale and boundary, service radius, and node layout; the second category is road traffic, which is composed of 5 secondary evaluation indicators, namely road network organization, entrance and exit settings, internal and external traffic conditions, walking and cycling systems, and parking spaces; and the third category is activity space, which is composed of six secondary evaluation indicators: square space, street space, leisure space, children's activity space, sports and fitness space, and courtyard space; the four categories are green spaces, which are respectively composed of three secondary evaluation indicators: plant configuration, space creation, and green space maintenance; the fifth category is supporting facilities, which are composed of four secondary evaluation indicators: landscape sketches, ground paving, barrierfree design, and later maintenance; and the sixth category is the humanistic spirit, which is composed of two secondary evaluation indicators of regional cultural characteristics and activity atmosphere [8], as shown in Figure 1.

## 4. Simulation Analysis of Landscape Architecture Evaluation Index Weight

The evaluation simulation of architectural landscape indicators adopts the construction environment evaluation of a certain city. In order to verify the validity of the proposed model, the experiment was carried out on the MATLAB platform with Windows 10 system whose running memory is 4 GB. The SPSS18.0 statistical software is used to fit 490 samples into the paper.

According to the descriptive statistics of key factor indicators, which are the standard value of the experiment, the experiment analysis is carried out. Moreover, experiments verify the significance of the index variables of landscape architecture data in the proposed model and compare the fit of the quality control model of landscape architecture.

### 4.1. Analysis of Evaluation Algorithm

As for the public space landscape evaluation index weight, *Y* represents the landscape architectural quality in the public space, that is, the target layer. *Y*^*I*^(*I*=1,2,…, *n*) refers to the first-level index level index, specifically the design process and construction process. *r*_*I*_ represents the importance of index *Y*^*I*^(*I*=1,2,…, *n*) to *Y*, that is, the weight of the landscape building quality parameters in the two evaluation index systems [9].

*y*_*m*_^*I*^ represents the *m* second-level evaluations under the first-level index level of landscape architecture quality evaluation, and *r*_*i*_^*I*^ indicates the importance of index *y*_*i*_^*I*^(*i*=1,2,…, *m*) to *Y*^*I*^, that is, the weight of index level *x*_*i*_^*I*^ [10].

Based on the mathematical principle of the analytic hierarchy process, the weight corresponding to the lower-level index *y*_*i*_^*I*^(*i*=1,2,…, *m*) of *Y*^*I*^(*I*=1,2,…, *n*) is determined. First, the lower-level indexes are compared two by two. After 10 comparisons, a judgment matrix describing the relative weight of *m* indexes is obtained, as shown in the following formula:

(1) $$\begin{array}{c} A^{I}=\left\{\begin{array}{cc} \left(r_{1}^{I}\right)/\left(r_{2}^{I}r_{1}^{I}\right)/\left(r_{2}^{I}\right) & \left(\left(r_{1}^{I}\right)/\left(r_{m}^{I}\right)\right)\\ \left(r_{2}^{I}\right)/r_{1}^{I}\left(r_{2}^{I}\right)/\left(r_{2}^{I}\right) & \left(r_{2}^{I}\right)/\left(r_{m}^{I}\right)\\ \left(r_{m}^{I}\right)/r_{1}^{I}\left(r_{m}^{I}\right)/\left(r_{2}^{I}\right) & \left(r_{m}^{I}\right)/\left(r_{m}^{I}\right) \end{array}\right\}={\left(a_{i}j^{I}\right)}_{m}xm, \end{array}$$

In formula (1): *a*_*i*_*j*^*I*^=1, *i*=*j* : *a*_*i*_*j*^*I*^=1, *i* ≠ *j* : *a*_*i*_*j*^*I*^(*a*_*i*_*q*^*I*^)/(*a*_*j*_*q*^*I*^), *i* ≠ *j* : *i*, *j*=1,2,…, *m*.

Simplify formula (1), then

(2) $$\begin{array}{c} A^{I}\cdot R^{I}=\left\{\begin{array}{cc} \left(r_{1}^{I}\right)/r_{1}^{I}\left(r_{1}^{I}\right)/\left(r_{2}^{I}\right) & \left(r_{1}^{I}\right)/\left(r_{m}^{I}\right)\\ \left(r_{2}^{I}\right)/r_{1}^{I}\left(r_{2}^{I}\right)/\left(r_{2}^{I}\right) & \left(r_{2}^{I}\right)/\left(r_{m}^{I}\right)\\ \left(r_{m}^{I}\right)/r_{1}^{I}\left(r_{m}^{I}\right)/\left(r_{2}^{I}\right) & \left(r_{m}^{I}\right)/\left(r_{m}^{I}\right) \end{array}\right\}=\left[\begin{array}{c} r_{1}^{I}\\ r_{2}^{I}\\ r_{m}^{I} \end{array}\right]\left[\begin{array}{c} mr_{1}^{I}\\ mr_{2}^{I}\\ mr_{m}^{I} \end{array}\right]=mR^{I}. \end{array}$$

According to the matrix theory, some important factors are highlighted when evaluating landscape building quality. Additionally, the judgment matrix contains the formula *A*^*I*^*R*^*I*^=*λ*_max_. Moreover, according to the largest eigenvalue of the judgment matrix *A*^*I*^ and the standardized eigenvector corresponding to *λ*_max_, the component *m* of *R*^*I*^ is the weight value of the index layer relative to *Y*^*I*^. When judging the order of the matrix A^I^, under the condition of the |*a*_*i*_*j*^*I*^|=∑_*q*=*l*_^*m*^*r*_*q*_^*I*^(*i*, *j*=1,2,…, *m*) × *Z*_*I*_*J* value, there is overall consistency [11]. Moreover, since the only largest feature root of A^I^ that is not 0 is *m*, it can be known that, under the premise that the weight value of each index is not clear, the relative weight value of the comparison index can be obtained through the pairwise comparison index, and the comparison judgment matrix can be generated [12].

In the two design parts of landscape architecture design and landscape concept design, the green concept is the main goal. Formula (2) uses an index vector to solve different component values.

Solving the quality of landscape architecture design is as follows:

(3) $$\begin{array}{c} \left|a_{i}j^{I}\right|=\sum_{q=l}^{m}r_{q}^{I}\left(i,j=1,2,\ldots ,m\right)\times Z_{I}J, \end{array}$$

(4) $$\begin{array}{c} \left|a_{i}j^{I}\right|=p\sum_{q=l}^{m}r_{q}^{I}\left(i,j=1,2,\ldots ,m\right)\times TZ_{I}J. \end{array}$$

In formula (4), *T* represents the effect of landscape design, $r_{i}^{I}=\left({\bar{r}}_{i}^{I}\right)/\sum_{q=1}^{m}\left(i=1,2,\ldots ,m\right)$ is the cost of landscape construction, and *p* refers to the effect of landscape completion. After standardizing the vector, we get

(5) $$\begin{array}{c} r_{i}^{I}=\frac{\left({\bar{r}}_{i}^{I}\right)}{\sum_{q=l}^{m}\left(i=1,2,\ldots ,m\right)}. \end{array}$$

The control formulas for the influencing factors of the landscape design concept and architectural design requirements are as follows:

(6) $$\begin{array}{c} V_{0}\left(k\right)=\frac{\left(r_{i}^{I}\right)}{Y^{I}\times \eta m}. \end{array}$$

In formula (6), *r* is the threshold value of influencing factors under unused conditions and *ηm* is *ηm*=1,2,…, *n*. The landscape building quality control formula process is obtained through the above process, which is the weight vector model of different indicators under the evaluation index *Y*^*I*^ [12].

### 4.2. Quality Index Analysis

The yaAHP analytic hierarchy process software is adopted, and quantification is performed based on the proportional scale. Moreover, the importance of indicators is sorted together to facilitate the construction of a judgment matrix. For the same level factors at the criterion level and the program level, a scale of 1–9 is used to compare and analyze the importance of different indicators, as shown in Table 2.

The weights of evaluation factors at all levels are ranked as follows. After the factor weight calculation, it can be seen in Table 3.

Among the first-level factors of public space landscape evaluation, landscape layout and road traffic have the highest weight values, which are 0.3034 and 0.2333. The two values are similar and far exceed other factors, while the lowest weight values are the supporting facilities and humanistic spirit, which are 0.0955 and 0.0622.

Among the secondary factors, the overall layout and parking space settings have the highest weight values, which are 0.1323 and 0.0901. The lowest weight value is the space construction index, which is 0.0156. The weight values of different factors can reflect the user's understanding of the index importance, and facilitate the case analysis of the postuse evaluation in the public space landscape environment, as shown in Table 4.

In Table 4, the calculation and analysis of the data are as follows. Among the evaluation indicators of landscape architecture based on the green concept, the parking space setting has the lowest score with a value of 1.88, which is followed by internal and external traffic conditions and regional characteristics with values of 2.05 and 2.17.

The statistics mentioned above show that the planning and setting of parking spaces is relatively unreasonable. Due to the large number of parking and mobile vehicles in the landscape area, the existing parking spaces cannot meet the overall demand [13]. Besides, there are no obvious characteristics in terms of regional characteristics, and the convergence is serious. However, the quality of the activity atmosphere, plant configuration, and overall layout is the highest, which are respectively 3.65, 3.64, and 3.50. Therefore, it can be seen that the overall atmosphere of the scenic spot is better, the overall layout is more reasonable, and the plant configuration is richer.

### 4.3. Comprehensive Score Calculated and Analyzed by Data

**Figure 2:**
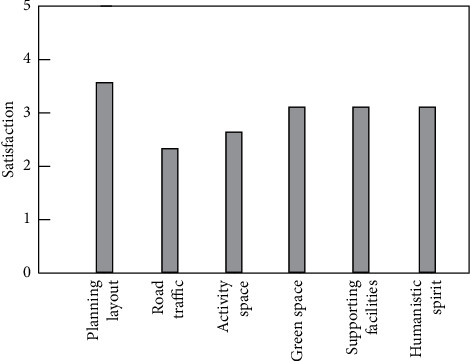
Satisfaction rating of the Level 1 index.

After data calculation and analysis, in the first-level evaluation index, the comprehensive evaluation data are shown in Table 5 and Figure 2.

**Figure 3:**
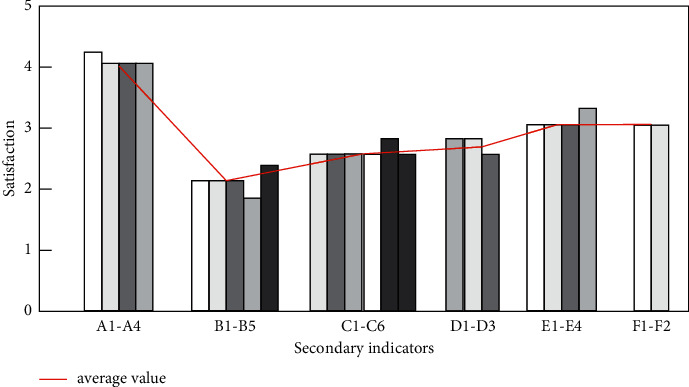
Satisfaction score of the Second-level index.

Adding the scores of the first-level evaluation indicators at all levels, the comprehensive score of the landscape space environment is 3.848 points, which shows that the overall situation is good and has great reference significance, but further improvement is still needed. The comprehensive index evaluation is shown in Figure 3.

In addition, the user's satisfaction with the overall landscape environment in this scenic spot is 3.84, of which the score in the humanistic spirit is relatively low, indicating that the regional characteristics and atmosphere need to be improved, while the overall satisfaction of the two major items of green space and supporting facilities is relatively high, which indicates that there are many things to learn from [14].

### 4.4. Evaluation Quality Comparison

The classification of the evaluation risk score assessment system is a multiclassification problem. When evaluating the multiclassification performance, it is necessary to decompose the multiclassification problem into multiple 2-classification problems, that is, L 2-classification problems [15]. There are several important indicators in the evaluation criteria of multiclassification.

  Let *N* be the total number of samples, and define the relevant indicators as follows:
  TP_*i*_ represents the *i*-th positive class of the correct evaluation;
  FN_*i*_ represents the positive class of error evaluation;
  FP_*i*_ represents the negative class of error evaluation;
  TN_*i*_ represents the correctly evaluated negative class.
  The proportion *w*_*i*_ of risk scores in the total sample is summed.

Finally, the model constructed by the random forest algorithm is evaluated through the four performance indexes of accuracy (A), weight-recall (*R*_*w*_), weight-precision (*P*_*w*_), and Kappa coefficient. *R*_*w*_ means to calculate the multiplication and summation of *R*_*i*_ and *w*_*i*_ of the i-th positive sample, respectively. The average is then calculated. This index represents the weighted average of the proportion of each evaluation risk score assigned correctly when it is judged to be positive and is used to measure the recognition ability of the model for each risk score. The calculation formula of *R*_*w*_ is

(7) $$\begin{array}{c} \left\{\begin{array}{c} R_{1}=\frac{{\text{TP}}_{1}}{{\text{TP}}_{1}+F_{1}},\\ R_{n}=\frac{\sum_{i=1}^{1},u_{1}}{L_{1}}. \end{array}\right) \end{array}$$

The optimal parameters of the evaluation system are selected through crossvalidation and grid search methods, and then the dataset is trained. The evaluation results are shown in Table 6.

The evaluation algorithm in this paper is superior to the random forest algorithm in the performance indicators, and the evaluation system can analyze the importance of the evaluation features and make more reasonable decisions.

## 5. Conclusion

Postevaluation is used as an entry point in the paper. The paper carries out quantitative data processing for building index evaluation, which mainly includes the following:

1. First, an evaluation index system is established, which uses analytic hierarchy and other methods to determine the weight of each evaluation factor.
2. Then, the evaluation is designed from both qualitative and quantitative aspects to explore and construct a set of public space landscapes that are suitable for the environmental evaluation system in cities.
3. Finally, a systematic and multilevel evaluation of the public space in the scenic spot is used to obtain a comprehensive evaluation result. Therefore, combining the theoretical research and simulation analysis of the green concept in the scenic spot, the design elements of the public space in the scenic spot, and the corresponding evaluation index are proposed. Meanwhile, the evaluation index system of the architectural design in the scenic spot is constructed.

There is still ample space for future exploration of landscape design. For example, the evaluation system and the selected evaluation methods for different types and different regions of open residential areas are different. The next step is to refine the quantitative indicators constructed by AHP to ensure the accuracy of the weight coefficient.

## Figures and Tables

**Table 1:** Evaluation index factors at all levels.

Target layer	Primary evaluation factor	Secondary evaluation factor
Landscape evaluation of public space in an open residential district	A planning layout	A1 overall layout
		A2 scale and boundary
		A3 service radius
		A4 node layout
	B Road traffic	B1 road network organization
		B2 entrance and exit settings
		B3 traffic conditions inside and outside
		B4 walking and cycling system
		B5 parking space setting
	C Event space	C1 square space
		C2 street space
		C3 leisure space
		C4 Children's activity space
		C5 sports fitness space
		C6 courtyard space
	D green space	D1 plant configuration
		D2 space creation
		D3 greenbelt conservation
	E supporting facilities	E1 landscape sketch
		E2 floor paving
		E3 barrierfree design
		E4 postmaintenance
	F Humanistic spirit	F1 regional cultural characteristics
		F2 event atmosphere

**Table 2:** Standardized values of evaluation indicators.

Factor *i* vs. factor *j*	Quantized value
Equally important	1
Slightly more important	3
Stronger important	5
Strongly important	7
Extremely important	9
The middle value of two adjacent judgments	2, 4, 6, 8

**Table 3:** First-level evaluation factor weight ranking.

Primary evaluation factor	Weights
Planning layout	0.3034
Road traffic	0.2333
Activity space	0.1904
Green space	0.1152
Supporting facilities	0.0955
Human spirit	0.0622

**Table 4:** Ranking of secondary evaluation factors.

Secondary evaluation factor	Weights
Overall layout	0.1323
Parking space setting	0.0901
Scale and boundary	0.075
Traffic conditions inside and outside	0.0573
Square space	0.056
Plant configuration	0.0515
Postmaintenance	0.0493
Service radius	0.0481
Node placement	0.0481
Street space	0.0415
Activity atmosphere	0.0415
Road network organization	0.0376
Entrance and exit settings	0.0284
Greenbelt conservation	0.0284
Barrierfree design	0.0268
Children's activity space	0.0264
Sports fitness space	0.0262
Courtyard space	0.0209
Regional cultural characteristics	0.0207
Ground paving	0.0202
Walking and cycling system	0.0199
Leisure space	0.0194
Landscape sketch	0.0189
Space creation	0.0156

**Table 5:** Comprehensive data of primary evaluation indicators.

Grade 1 index	Satisfaction	Weights	Score
Planning layout	4.23	0.2002	1.04
Road traffic	2.31	0.2123	0.54
Activity space	2.75	0.1782	0.67
Green space	2.96	0.1284	0.81
Supporting facilities	3.01	0.1244	0.27
Humanistic spirit	2.97	0.1565	0.17

**Table 6:** Comparison between the proposed algorithm and the random forest algorithm.

Model category	*A*	*R*_*w*_	*P*_*w*_	Kappa coefficient
Algorithm	0.977	0.984	0.962	0.948
Random forest	0.907	0.935	0.912	0.903

## Data Availability

The data used to support the findings of this study are available from the corresponding author upon request.

## References

[1] Adnan J. N. (2021). Diagnosing the causes of poor quality management in IraqiConstruction projects using technique of root cause analysis. *IOP Conference Series: Materials Science and Engineering*.

[2] Fang J., Partovi F. Y. (2020). Criteria determination of Analytic hierarchy process using a topic model. *Expert Systems with Applications*.

[3] Fk A., Ab B., Jas B. (2020). Mineral processing plant site selection using integrated fuzzy cognitive map and fuzzy analytical hierarchy process approach: a case study of gilsonite mines in Iran. *Minerals Engineering*.

[4] Zhu C., Li N. (2017). Study on grey clustering model of indoor air quality indicators. *Procedia Engineering*.

[5] Chen P. (2020). Effects of the entropy weight on TOPSIS. *Expert Systems with Applications*.

[6] Zhang H., He X., Mitri H. (2019). Fuzzy comprehensive evaluation of virtual reality mine safety training system. *Safety Science*.

[7] Xin L., Junjie Y., Hongcai Y., Hongcai C. (2017). Study on evaluation index system of green mine construction. *IOP Conference Series: Earth and Environmental Science*.

[8] Islam T., Islam R., Pitafi A. (2021). The impact of corporate social responsibility on customer loyalty: the mediating role of corporate reputation, customer satisfaction, and trust - ScienceDirect. *Sustainable Production and Consumption*.

[9] Suárez-Eiroa B., Fernández E., Méndez-Martínez G., Soto-Oñate D. (2019). Operational principles of circular economy for sustainable development: linking theory and practice. *Journal of Cleaner Production*.

[10] Wei P., Wulin P., Cheng H. (2019). Assessing the green economy in China: an improved framework. *Journal of Cleaner Production*.

[11] Weber H., Weber M. (2020). When means of implementation meet Ecological Modernization Theory: a critical frame for thinking about the Sustainable Development Goals initiative. *World Development*.

[12] Severo E. A., Guimarães J. C. F. d., Dorion E. C. H., Nodari C. H. (2015). Cleaner production, environmental sustainability and organizational performance: an empirical study in the Brazilian Metal-Mechanic industry. *Journal of Cleaner Production*.

[13] Wang Z., Zhou S., Wang Mo, Zhang D. (2020). Cost-benefit analysis of low-impact development at hectare scale for urban stormwater source control in response to anticipated climatic change. *Pubmed*.

[14] Alawneh G., Ali A. (2019). A new index for assessing the contribution of energy efficiency in LEED 2009 certified green buildings to achieving UN sustainable development goals in Jordan. *Taylor & Francis*.

[15] Ali A., Alsaqoor S. (2019). Energy efficient of using chilled water system for sustainable health care facility operating by solar photovoltaic Technology. *Elsevier*.

